# Supplementary material for: The interdomain flexible linker of the polypeptide GalNAc transferases dictates their long-range glycosylation preferences
Source: Nat Commun. 2017 Dec 5;8:1959. doi: 10.1038/s41467-017-02006-0 (PMC5716993; doi:10.1038/s41467-017-02006-0)
Supplement: Supplementary file 1 — Supplementary Information [file 41467_2017_2006_MOESM1_ESM.pdf]

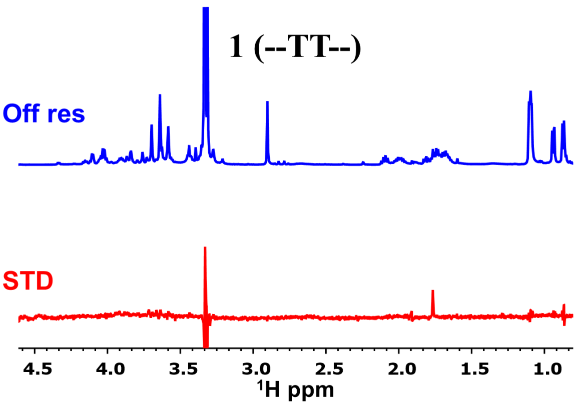

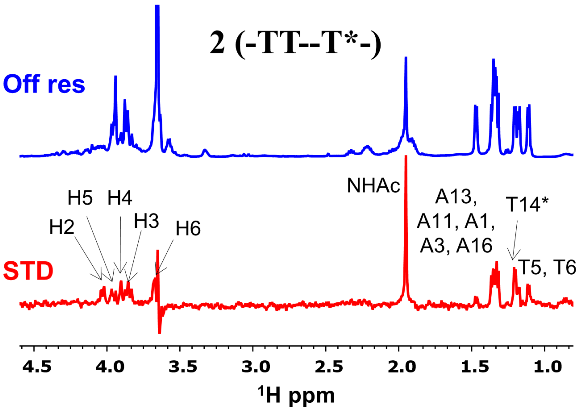

Proton	% STD relative
H2	100
H3	<20
H4	49
H5	<20
H6	<20
NHAc	45
A13, A11, A1, A3, A16	<20
T14*	21
T5, T6	<20

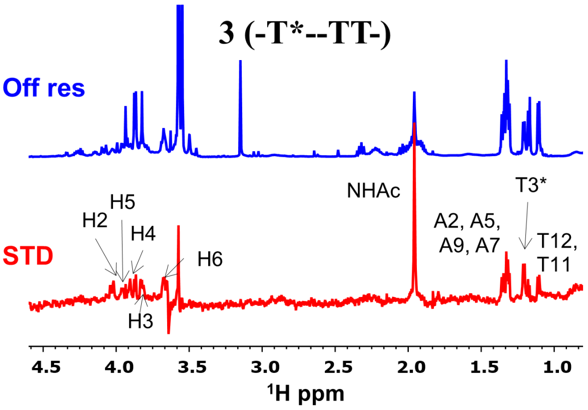

Proton	% STD relative
H2	100
H3	75
H4	93
H5	30
H6	29
NHAc	87
A2, A5, A9, A7	<20
T3*	31

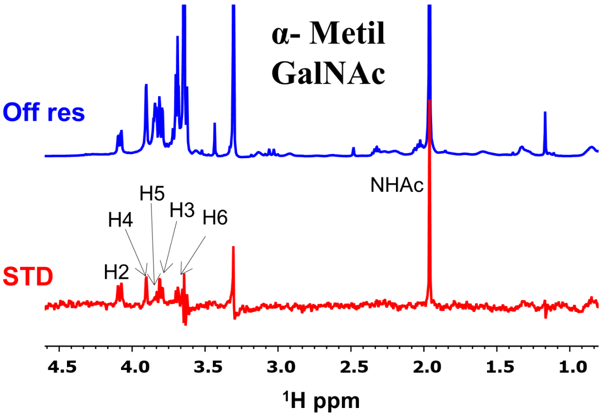

Proton	% STD relative
H2	100
H3	55
H4	42
H5	25
H6	<20
OMe	<20
NHAc	46

Supplementary Figure 1. STD-NMR Experiments. For all compounds the reference spectrum (Off res) is displayed in blue color while the STD spectrum (STD) is displayed in red. No STD response was observed in the spectrum with peptide **1**. For the other compounds (monoglycopeptides **2**, **3** and α-methyl-GalNAc) the key proton resonances are marked in each STD spectrum and their relative STD percentages are represented in the table at right of the spectra.

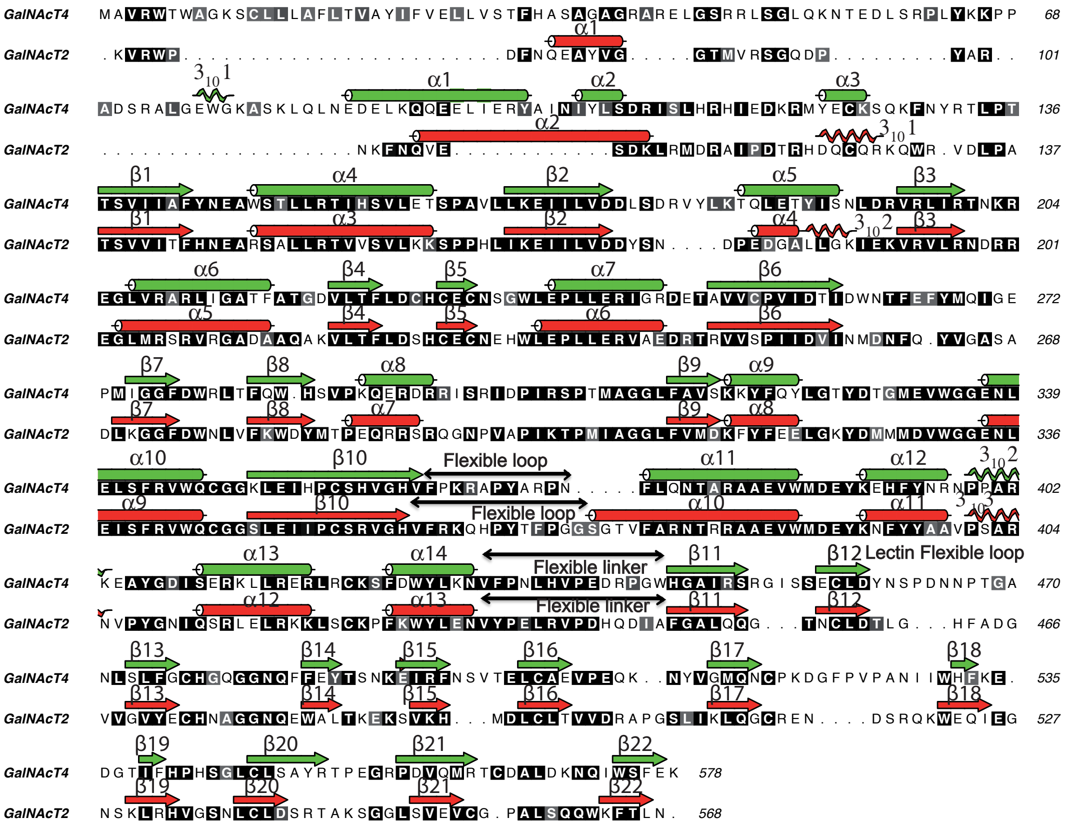

Supplementary Figure 2. Sequence alignment of human GalNAc-T2 and GalNAc-T4. Secondary structure elements from the GalNAc-T2 and T4 structures are shown, with α-helices, 3_{10}-helices and β-strands in red and green, respectively. The regions encompassing the flexible loop and linker are indicated with double sided arrows.

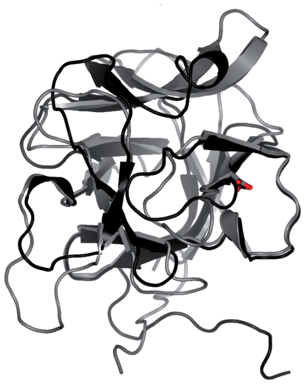

Supplementary Figure 3. Superposition analysis between GalNAc-T2 (black) and GalNAc-T4 (grey) lectin domains. Asp458 and Asp459 are shown as black and grey carbon atoms, respectively.

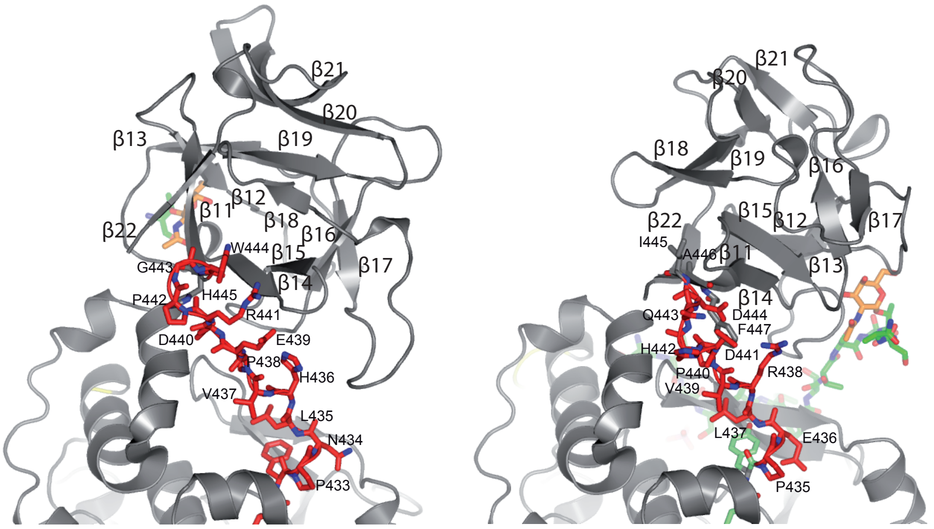

Supplementary Figure 4. Close up-view of the GalNAc-T4 (left) and GalNAc-T2 (right) lectin domains. Colours for the peptides, proteins, and the GalNAc moiety are the same as indicated in **Fig. 3**. The flexible linkers are shown as sticks in red carbon atoms. The numbering of the lectin domain secondary structural elements is also shown.

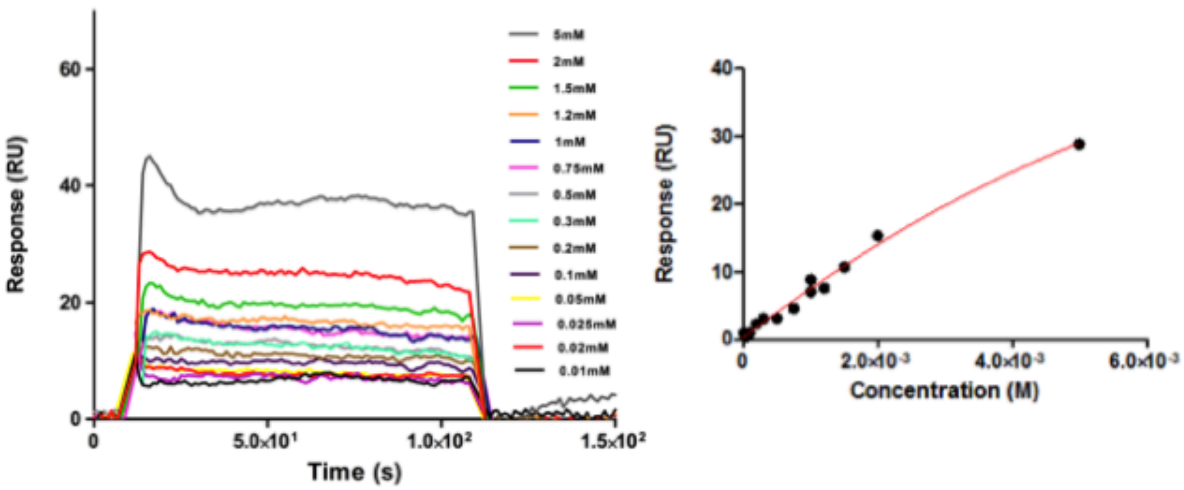

Supplementary Figure 5. SPR sensogram. (left) Sensogram for binding of the monoglycopeptide **3** to GalNAc-T4**.** Used ligand concentrations are reported in the inset legend. (right) Fitting of SPR data. The end-points of the various injections were plotted against protein concentration. Note that the Kd could not be determined because binding saturation could not be achieved.

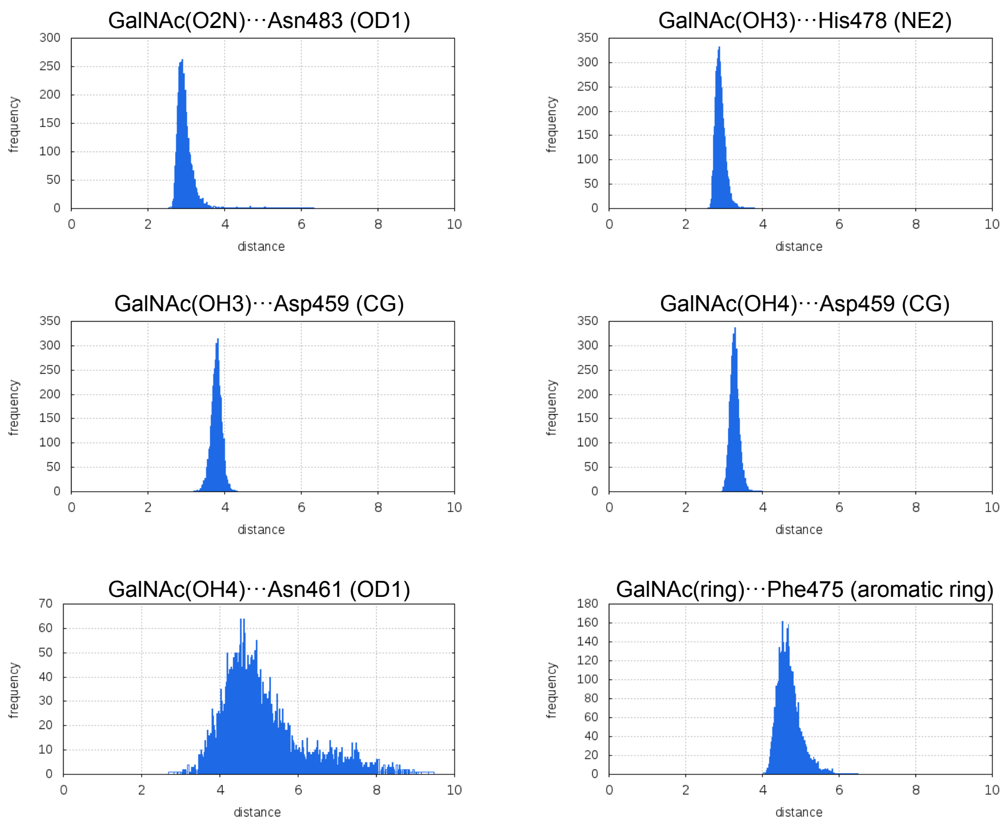

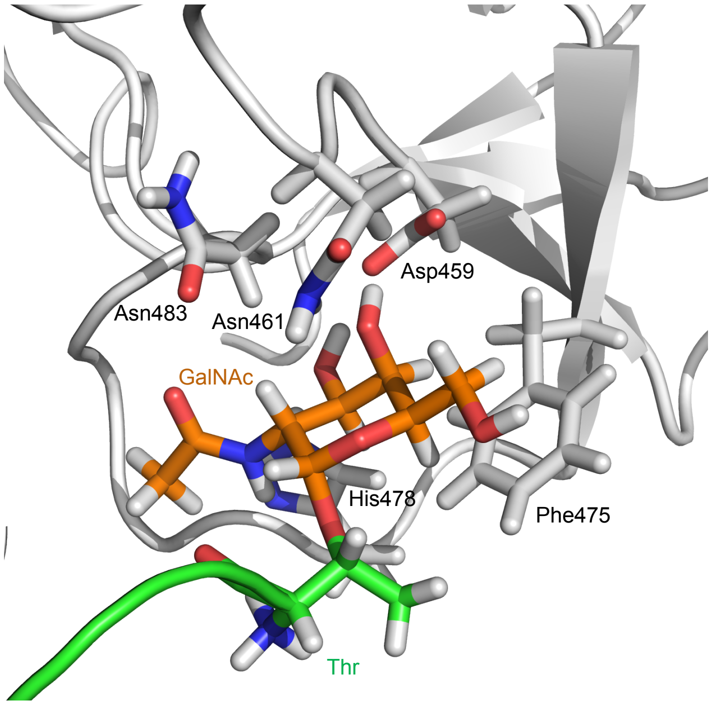

Supplementary Figure 6. Representative distance distributions obtained by 200 ns MD simulations on the complex between GalNAc-T4 and glycopeptide 3. These data indicate that the GalNAc unit is retained in the lectin domain during the simulation time.

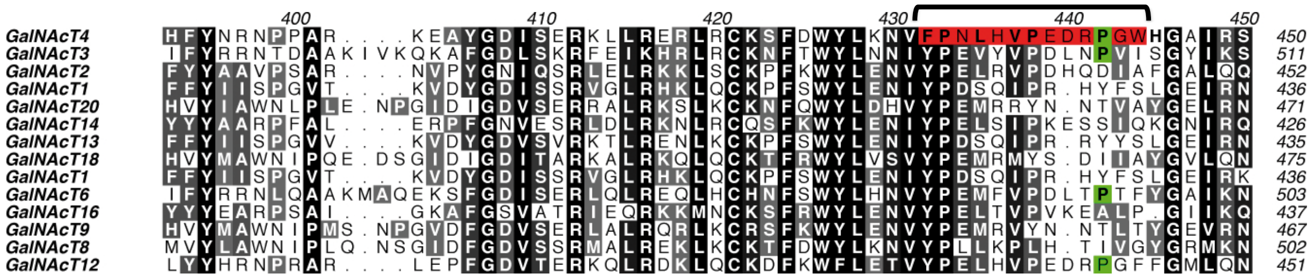

Supplementary Figure 7. Multiple sequence alignment for GalNAc-Ts spanning the regions around the flexible linker. The sequence highlighted in red denotes the flexible linker of GalNAc-T4. The additional Pro in GalNAc-T4/T3/T6/T12 is highlighted in green. The alignment shows the most conserved region between the flexible linkers is towards the N-termini whereas more dissimilarities are around the C-termini.

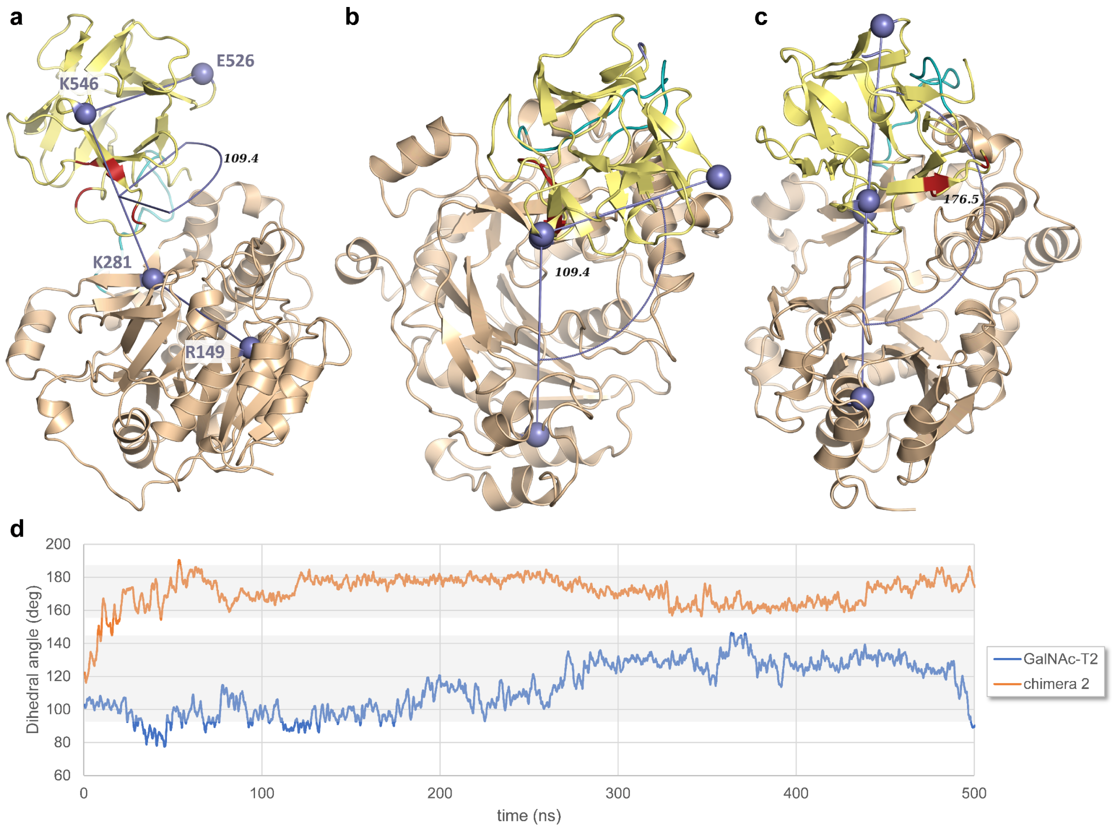

Supplementary Figure 8. Rotation of the lectin domains in GalNAc-T2 and chimera 2. **a**, Selected positions (alpha carbons, in blue) arbitrarily chosen to define a dihedral angle describing the relative positions of the lectin and catalytic domains. **b**, Top view of the first MD snapshot (time = 0 ns) showing a nearly perpendicular relative orientation (109º) of the two domains in chimera2. **c**, Top view of the last MD snapshot (time = 500 ns) showing a nearly parallel relative orientation (177º) of the two domains in chimera 2. **d**, Evolution of the rotation dihedral angle for GalNAc-T2 (in blue) and chimera 2 (in orange) as a function of the MD simulation time. For chimera 2, a quick ~70º rotation of the lectin domain is observed within the first ~30 ns, remaining stable in a nearly parallel orientation for the rest of the simulation. However, for GalNAc-T2, the initial close to perpendicular relative disposition of the domains is maintained, with fluctuations, throughout all the simulation.

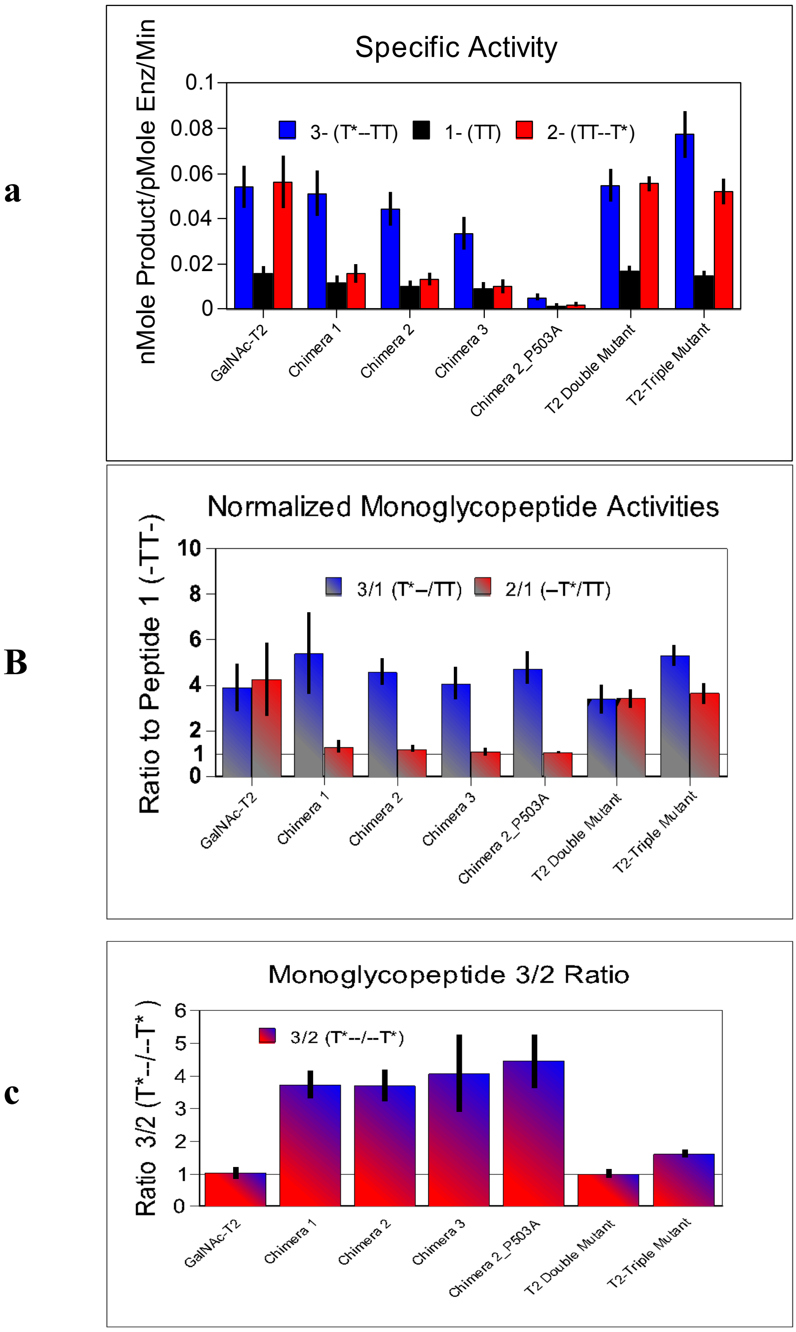

.

Supplementary Figure 9. Specific activity of the GalNAc-T2 chimeras and mutants and their selected substrate activity ratios. a, Plot of specific activity calculated as described in the **Methods** from 3-5 (except 2 for chimera 2P503A) independent time course reactions. **b**, Plot of monoglycopeptide specific activity normalized to the nonglycosylated substrate , i.e. peptide **3**/**1** and **2**/**1**. **c**, Plot of the ratio of monoglycopeptide **3** over monoglycopeptide **2**, i.e. peptide **3**/**2**. The latter provides an indication of the selectivity of the transferase for the 2 substrates. Black lines represent obtained standard deviations as described in the **Methods**.

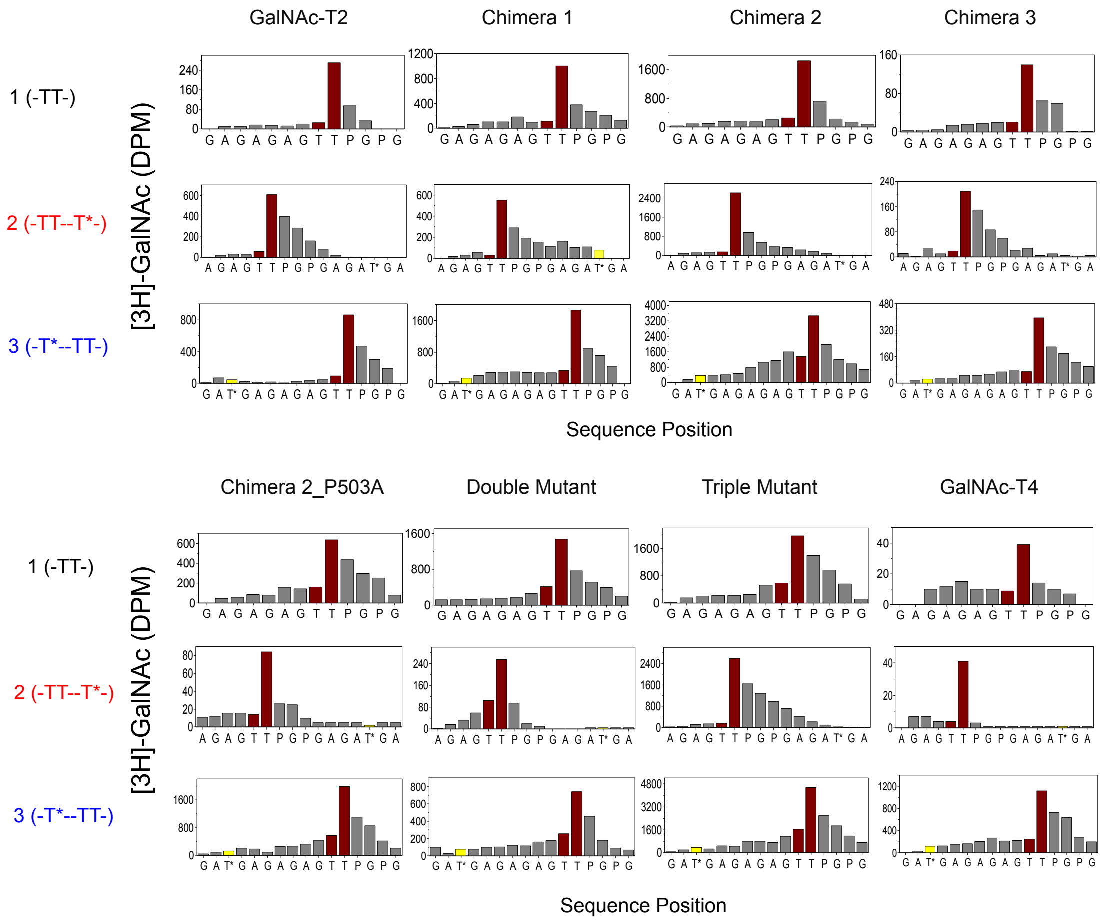

Supplementary Figure 10. Sites of [3H]-GalNAc incorporation into substrates 1-3 by GalNAc-T2 and its chimeras and mutants as determined by Edman amino acid sequencing. Each Edman sequencing cycle was collected and its [^{3}H]-GalNAc content determined by scintillation counting as described in the **Methods**. The bars represent the [^{3}H]-GalNAc content determined at each cycle, the dark red bars represent the two potential Thr acceptor sites, the yellow bar represents the site of prior GalNAc-Thr glycosylation (T*) and the grey bars denote the remaining residues. Sequencing was occasionally terminated a few cycles after the last potential acceptor Thr. See **Methods** for further details.

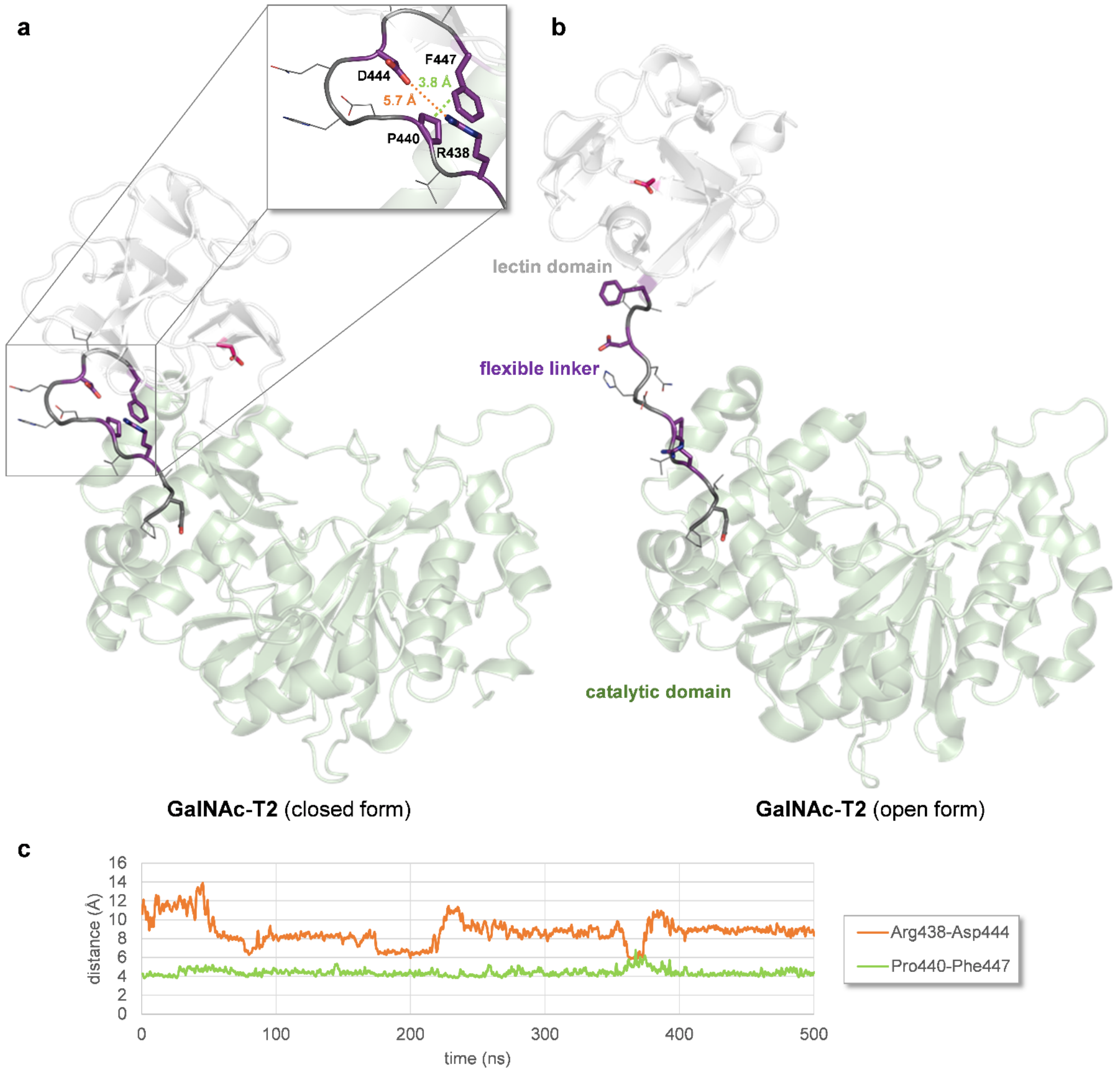

Supplementary Figure 11. a-b, Structure and dynamics of GalNAc-T2 flexible linker (in purple) connecting the catalytic (in pale green) and lectin (in grey) domains. The X-ray structures of both the closed (PDB entry 5AJP) and open (PDB entry 2FFU) forms of the protein are used as references. **c**, Key polar and van der Waals interactions responsible for the folded conformation of the flexible peptide monitored throughout MD simulations in explicit water. CH–π interactions (in green) are tightly maintained along the whole simulation; salt-bridge (in orange) fluctuates more but stays in the attractive range most of the simulation time.

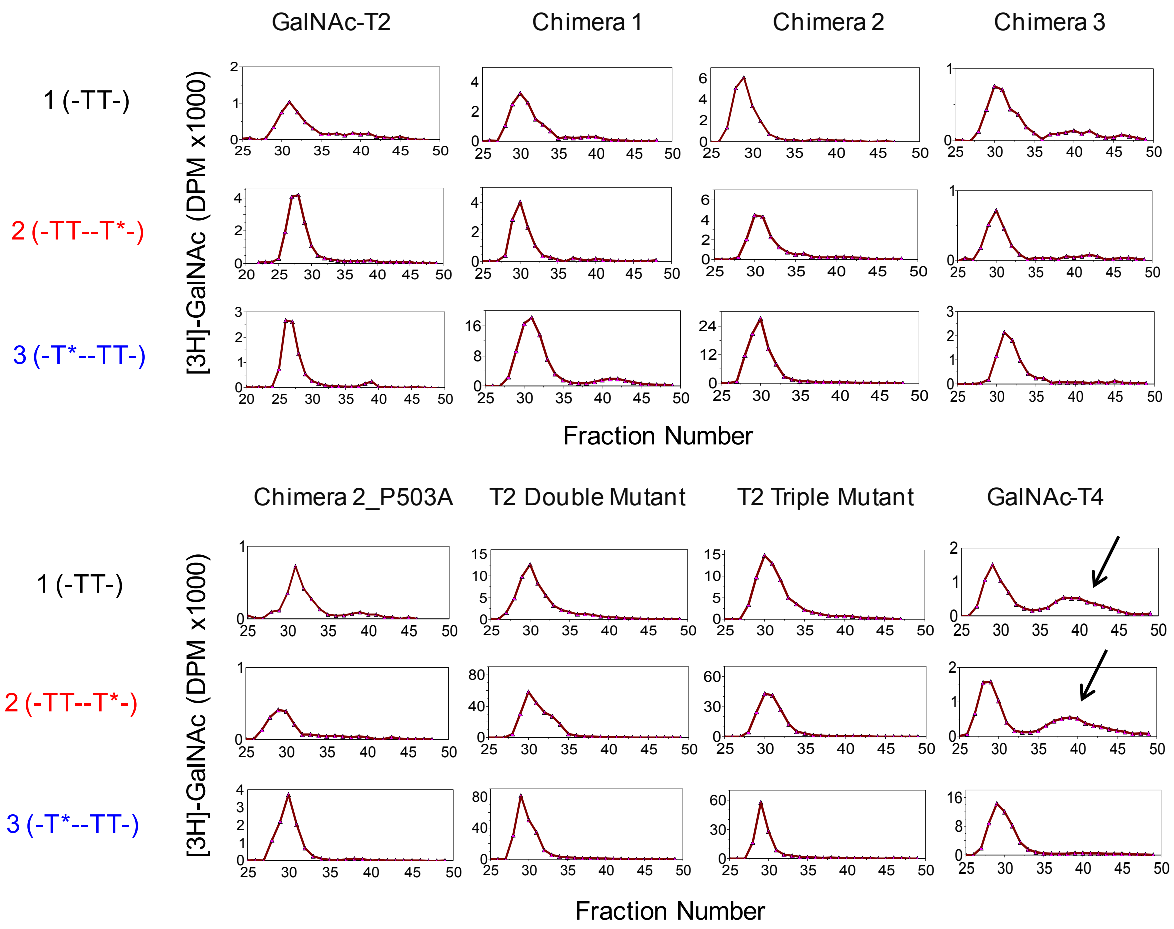

Supplementary Figure 12. Sephadex G10 gel filtration chromatograms of the [^{3}H]-GalNAc products of GalNAc-T2, its chimeras and mutants, and GalNAc-T4. [^{3}H]-GalNAc content at each fraction was measured by scintillation counting. The first peak of [^{3}H]-GalNAc content (fractions ~20-30) represents glycosylated substrate (glyco)peptide, while the second peak (arrow) represents the hydrolysis product free [^{3}H]-GalNAc. See **Methods** for further details.

Supplementary Table 1. Data collection and refinement statistics. Values in parentheses refer to the highest resolution shell. Ramachandran plot statistics were determined with PROCHECK.

	**GalNAc-T4 in complex with monoglycopeptide 3***
**Space group**	P1
**Wavelength (Å)**	0.97
**Resolution (Å)**	20-1.90 (2.00-1.90)
**Cell dimensions**<br>**a, b, c (Å)**<br>**α, β, γ (°)**	*a = 65.45*<br>*b*= 79.88<br>*c* = 88.61<br>*116.47, 96.74, 104.72*
**Mn(I) half-set correlation CC(1/2)**	0.988 (0.560)
**Unique reflections**	115064
**Completeness**	97.5 (95.8)
R_{pim}	0.068 (0.556)
$I/\sigma(I)$	6.9 (1.3)
**Redundancy**	3.6 (3.2)
R_{work} / R_{free}	0.225/0.273
**RMSD from ideal geometry, bonds (Å)**	0.014
**RMSD from ideal geometry, angles (°)**	1.718
**<B> GalNAc-T4 (Å^2)**	32.48
**<B> GalNAc (Å^2)**	48.44
**<B> Thr3-GalNAc (Å^2)**	52.45
**<*B*> solvent (Å^2)**	43.22
**<*B*> Ethylenglycol (Å^2)**	48.40
**<*B*> Glycerol (Å^2)**	57.74
**Ramachandran plot:**	
**Most favoured (%)**	96.71
**Additionally allowed (%)**	2.89
**Disallowed (%)**	0.40
**PDB ID**	5NQA

*Note that the density is only good enough to model either GalNAc or GalNAc bound to Thr3 of monoglycopeptide **3**.

Supplementary Table 2. Michaelis-Menten Kinetic Parameters for GalNAc-T2, GalNAc-T2-Triple Mutant, GalNAc-T2 Chimera 3, and GalNAc-T4 against (glyco)peptide substrates 1, 2 and 3. Parameters were obtained from the nonlinear fit of the data plotted in **Fig. 2a** and **4c** using GraphPad Prism 7.03.

**Transferase**	**Kinetic parameters**	**(glyco)peptide substrate**		
		**1(--TT--)**	**2(-TT—T*-)**	**3(-T*--TT-)**
GalNAc-T2[1]	Vmax[2]	0.71 ± 0.09	3.12 ± 0.07	3.79 ± 0.34
	Km[3]	1.29 ± 0.25	0.18 ± 0.01	0.75 ± 0.13
	**Catalytic efficiency**[4]	**0.56 ± 0.2**	**17.0 ± 2.3**	**5.03 ± 1.8**
Triple Mutant	Vmax	1.06 ± 0.35	1.80 ± 0.06	2.82 ± 0.10
	Km	3.28 ± 1.55	0.40 ± 0.04	0.57 ± 0.05
	**Catalytic efficiency**	**0.33 ± 0.3**	**4.54 ± 0.9**	**4.94 ± 0.9**
Chimera 3	Vmax	0.80 ± 0.08	1.35 ± 0.11	3.69 ± 0.29
	Km	1.02 ± 0.21	0.70 ± 0.14	0.99 ± 0.16
	**Catalytic efficiency**	**0.79 ± 0.3**	**1.93 ± 0.8**	**3.73 ± 1.2**
GalNAc-T4[5]	Vmax	-	-	3.09± 0.25
	Km	-	-	0.041 ± 0.018
	**Catalytic efficiency**	**-**	**-**	**75.2 ± 34**

1) Note that the GalNAc-T2 kinetic data was analyzed by GraphPad Prism's 1/y weighting feature that improved the concordance of the fit for peptide 3 at high substrate concentrations, i.e its Vmax value. Parameters for peptides 1 and 2 were unchanged using this weighting compared to the unweighted fit.
2) V_{max} units: mM/(nmole*min), with standard deviation.
3) K_m units: mM, with standard deviation.
4) Catalytic efficiency (V_{max}/K_m): $(nmole*min)^{-1}$. Note that the given errors were estimated from the largest percent error of the V_{max} or K_m value.
5) For GalNAc-T4 the activities of peptides 1 and 2 were too low to determine their kinetic constants.
