# Supplementary material for: The interdomain flexible linker of the polypeptide GalNAc transferases dictates their long-range glycosylation preferences
Source: Nat Commun. 2017 Dec 5;8:1959. doi: 10.1038/s41467-017-02006-0 (PMC5716993; doi:10.1038/s41467-017-02006-0)
Supplement: Supplementary file 3 — Descriptions of Additional Supplementary Files [file 41467_2017_2006_MOESM3_ESM.pdf]

**Descriptions of Additional Supplementary Files**

File Name: Supplementary Movie 1
Description: Molecular dynamic simulations of the GalNAc-T4 in complex with UDP/Mn+2 and monoglycopeptide 3.

File Name: Supplementary Movie 2
Description: Molecular dynamic simulations of the GalNAc-T2.

File Name: Supplementary Movie 3
Description: Molecular dynamic simulations of the GalNAc-T4.

File Name: Supplementary Movie 4
Description: Molecular dynamic simulations of the chimera 2.

File Name: Supplementary Movie 5
Description: Molecular dynamic simulations of the Arg397 and Glu487 interaction present in the GalNAc-T2 flexible linker.

File Name: Supplementary Movie 6
Description: Molecular dynamic simulations of the GalNAc-T2 flexible linker.

File Name: Supplementary Movie 7
Description: Molecular dynamic simulations of the GalNAc-T3 flexible linker

File Name: Supplementary Movie 8
Description: Molecular dynamic simulations of the double mutant.

File Name: Supplementary Movie 9
Description: Molecular dynamic simulations of the triple mutant. - We are happy with the editor´s summary.
